# A Passing Race

**Published:** 1917-02

**Authors:** 


**A Passing Race,** J. G. B. Bulloch, Washington, Western Med. Times, Nov. This is a plea for the saving of the old American stock, from its present tendency to die out as the result of 1. Intellectual Overdevelopment, 2. Over Strenuosity, 3. Sexual Disease, 4. Diversion of the Mind, 5. Decline of the Religious Spirit, 6. Climatic Influences.

The sole complimentary reason for the decline of the unhyphenated American is retracted, in the statement that some of the old families have maintained their prestige. In exactly the same spirit that we would protest against an aspersion against Jews, German-Americans or any other part of our composite population, we wish to enter a plea against this sort of article which recurs every year or two, in one form or another, in sociologic and medical literature. First of all, the colonial Americans do not constitute a race. They are not entirely or even mainly English or even British, as war disputants—though not Dr. Bulloch—assume. All four of the British nationalities were represented in considerable numbers in the early migration to America, as well as at least five continental nations. The Jews have recently celebrated the 250th anniversary of the arrival of the first of their race on American soil and individuals from many other races were early present. Quite aside from the fact that European nationality by no means implies a distinct racial development, practically all colonial Americans are of mixed blood so far as these nationalities are concerned, whereas the more recent migrants have often, perhaps usually, preserved a single national strain down to the present generation. The English themselves are a very much mixed race and have had a considerable immigration, of peaceable nature, subsequent to the last conquest by the Normans. Even if the colonial Americans had been entirely British, they would have represented four quite distinct nationalities, the Irish, Scotch and Welch fairly established racially, the English composed of at least five different strains, politically significant additions to the melting pot having ceased just twice as far back from the beginning of colonization of ''New England'' as the latter is from the present.

But call the colonials a race or not, they are not passing. Almost any of the last six or seven decades has deposited in America from one to three times the total population reached by the colonial Americans at the time of the organization of the country after the Revolution. In spite of this, the latter still amount to just about half of the present population. About the maximum human prolificity is a multiplication by three for a generation—that is to say, an average of 12 children. half girls and half boys, half of the latter surviving childhood and adolescence, according to conditions present up to a comparatively few years ago. We know of one family whose male, adult members five generations from the original immigrant numbered within a very few of 243, which is the fifth power of 3. This family, in spite of division of political sentiment, furnished over 60 men for the continental army and over 300 for the Civil War. Another family, whose progenitors came over slightly earlier, indeed on the Mayflower, had 298 men in the Revolution. A descendent was unable to secure membership in the Sons of the Revolution on account of an abundance of riches. Some 20 men of the exact name as his great grandfather were on the Revolutionary rolls and it was impossible to demonstrate just which one of these was the particular ancestor sought. There are literally, hundreds of different family surnames, each representing descent from a single colonial immigrant or from two or more brothers, and each now enrolling 1000 or more descendents, listed by name. No people can hold its rank proportionately by the tedious process of child bearing—at the standard rate of 3.5% per annum—and child rearing—at the standard rate of 20 to 25% mortality before maturity is reached—against the importation of a foreign ingredient varying from 1 to 4% per annum and itself equally capable of reproduction. But the colonial American is not "passing," any more than the American Indian whose numbers are, at present, as great for New York State as in the seventeenth century and whose numbers for the whole country or the whole continent are probably as great as at Columbus's first visit. On the contrary, the colonial American is multiplying at the normal rate for any established people though undoubtedly not as fast as he did in the pioneer period nor as fast as introduced stock multiplies in the corresponding period following transplantation to a fresh soil.

